# Supplementary material for: Defective ribosomal products challenge nuclear function by impairing nuclear condensate dynamics and immobilizing ubiquitin
Source: EMBO J. 2019 Jul 4;38(15):e101341. doi: 10.15252/embj.2018101341 (PMC6669919; doi:10.15252/embj.2018101341)
Supplement: Supplementary file 1 — Appendix [file EMBJ-38-e101341-s001.pdf]

APPENDIX FILE

Defective ribosomal products (DRiPs) challenge nuclear function by impairing nuclear condensate dynamics and immobilizing ubiquitin

Laura Mediani[1], Jordina Guillén-Boixet[2], Jonathan Vinet[1], Titus M. Franzmann[2], Ilaria Bigi[1], Daniel Mateju[2], Arianna D. Carrà[1], Federica F. Morelli[1], Tatiana Tiago[1], Ina Poser[2], Simon Alberti[2], Serena Carra[1,3,*]

TABLE OF CONTENTS

Appendix Figure Materials and Methods

Appendix Figures

Appendix Figure References

EXPANDED VIEX AND APPENDIX FIGURE MATERIALS AND METHODS

RT-qPCR

Total RNA was isolated from HeLa cells treated as indicated in the figure legends, using the ReliaPrep™ RNA Miniprep Systems (Promega) according to the manufacturer's instruction. 1 µg of RNA was reverse transcribed using random hexamer and Superscript III reverse transcriptase (Thermo Fisher), following manufacturer's instruction. PCR amplification was performed using Maxima SYBR Green qPCR Master Mix polymerase (Thermo Fisher). Cyclophilin mRNA was used as internal control for ribosomal RNA processing (REF Kwon et al Science). RPL0 was used as internal control for HSPA1A mRNA analysis. Primers used for PCR analysis were as follow: pre-rRNA 45S-for (5'- GAACGGTGGTGTGTCGTTC-3'); pre-rRNA 45S-rev (5'-GCGTCTCGTCTCGTCTCACT-3') (Nishimura, Kumazawa et al., 2015); precursor 18S-5'-junction-for (5'-GCCGCGCTCTACCTTACCTACCT-3'); precursor 18S-5'-junction-rev (5'-CAGACATGCATGGCTTAATCTTTG-3'); precursor 5.8S-5'-junction-for (5'-TACGACTCTTAGCGGTGGATCA-3'); precursor 5.8S-5'-junction-rev (5'-TCACATTAATTCTCGCAGCTAGCT-3'); 5.8S-for (5'-ACTCGGCTCGTGCGTC-3'); 5.8S-rev (5'-GCGACGCTCAGACAGG-3'); cyclophilin-for (5'-TGCCATCGCCAAGGAGTAG-3'); cyclophilin-rev (5'-TGCACAGACGGTCACTCAAA-3') (Kwon, Xiang et al., 2014); 18S-for (5'-CTGCCCTATCAACTTTCGATGGTAG-3'); 18S-rev (5'-CCGTTTCTCAGGCTCCCTCTC-3'); 28S-for (5'-TGTCGGCTCTTCCTATCATTGT-3'); 28S-rev (5'-ACCCAGCTCACGTTCCCTATTA-3'); HSPA1A-for (5'-GCTTCAAGACTTTGCATTTCC-3'); HSPA1A-rev (5'-AAAGCAAGTTCAGTACTTCACC-3'); RPL0-for (5'-TTAAACCCTGCGTGGCAATCC-3'); RPL0-rev (5'-CCACATTCCCCCGGATATGA-3').

Analysis of puromycylated protein degradation

For the analysis of nuclear puromycylated protein degradation, HeLa cells were treated with puromycin (10 µg/ml) for 1 hr, followed by recovery in drug-free medium or in presence of MG132 (10 µM) or ammonium chloride (20 mM). 6 h after recovery, cytosolic and nuclear proteins were fractionated as previously described (Morelli, Verbeek et al., 2017). Puromycylated protein accumulaton in the nuclear fractions was analyzed by SDS-PAGE followed by immunoblotting.

Immunoprecipitation of puromycylated proteins from nucleoplasmic and nucleolar extracts

Nucleoli were isolated as described in the main materials and methods. Nucleoli resuspended in S2 solution were pelleted by centrifugation at 1430 xg and 4°C for 5 min. The resulting nucleolar pellet was lysed with 22 µl of 4% SDS, 50 mM Tris-HCl pH 8 and let 10 min at room temperature. After, RIPA buffer composition (50 mM Tris-HCl pH 8, 150 mM NaCl, 1 mM $MgCl_2$, 1% Triton X-100, 0.1% SDS, 1 mM EDTA, EDTA-free protease inhibitors) was reconstituted in a final volume of 900 µl. Nucleic acids were digested with 1.5 U of Benzonase endonuclease (in-house) during 30 min at 4°C. Nucleolar lysates were centrifuged at 18000 xg and 4°C for 5 min and the supernatant was kept for subsequent immunoprecipitation.
450 µl of nucleolar extract (135 µg of protein) or 450 µl of diluted nucleoplasmic fraction (nucleoplasmic fraction diluted 1/3 to obtain a final RIPA buffer composition) were pre-cleared with 20 µl of Dynabeads protein A (Invitrogen) for 1 h at 4°C. The resulting pre-cleared extracts were incubated O/N at 4°C with 10 µg of anti-Puromycin antibody covalently cross-linked to 50 µl of Dynabeads protein A. Immunoprecipitates were washed 3 times with RIPA buffer and eluted with Laemli sample buffer by heating at 65°C for 20 min. After, proteins were resolved in 4-12% NuPAGE Bis-Tris protein gels (Thermo Fisher Scientific) and analyzed by western blotting (anti-Ubiquitin antibody P4D1).

Chemicals, Primary and Secondary antibodies

Chemicals: Amylo-Glo (TR-300-AG, Biosensis); Anisomycin (A9789, Sigma-Aldrich); Cycloheximide (C7698, Sigma-Aldrich); Crystal violet (C0775; Sigma-Aldrich); DAPI (SC3598, Santa Cruz Biotechnology); Eeyarestatin I (E1286; Sigma-Aldrich); Ivermectin (sc-203609A, Santa Cruz Biotechnologies); O-Propargyl-puromycin (OP-puro; NU-931-05, Jena Bioscience); Puromycin (P8833, Sigma-Aldrich); VER-155008 (SML0271, Sigma-Aldrich); Z-Leu-Leu-Leu-al (MG132; C2211, Sigma-Aldrich).
Primary antibodies: Flag (Anti-FLAG M2; F1804, Sigma-Aldrich); Fibrillarin (G-8; sc-374022, Santa Cruz Biotechnology); FUS (sc-47711, Santa Cruz Biotechnology); HA (H9658; Sigma-Aldrich); H2A (PA5-35893, Thermo Fisher); H2A-Ub (E6C5; 05-678, Merck); HSPA1A (Hsp70/Hsc70; SMC-104A, StressMarq Biosciences); HSPA8 (Hsc70/Hsp73; SMC-151A, StressMarq Biosciences); LMNB1 (sc-6217, Santa Cruz Biotech); Proteasome 20S alpha 1+2+3+5+6+7 (ab22674, Abcam); PML (for immunofluorescence, Ab53773, Abcam and SC-5621, Santa Cruz Biotechnology); polyUb proteins (FK1; BML-PW8805-0500, Enzo); Puromycin (MABE343, Merck Millipore); SC-35 (S4015, Sigma-Aldrich); TUBA4A (T6074, Sigma-Aldrich); alpha-Tubulin (DM1a, in house); Ubc9 (Ab30505, Abcam); Ubiquitin (Z0458, Dako and P4D1, Santa Cruz); Ubiquityl-Histone-H2A (clone E6C5; 05-678, Merck); VCP (MA3-004, Thermo Scientific); 53BP1 (PA116565, Millipore).
Secondary antibodies: donkey anti-goat IgG-HRP (SC-2020, Santa Cruz Biotech); mouse IgG HRP linked whole ab (NXA931, GE Healthcare); rabbit IgG HRP linked whole ab (NA934, GE Healthcare); Alexa Fluor™ 594 Azide (A-10270, Thermo Scientific); Goat anti-Mouse IgG, IgM (H+L), Alexa Fluor® 488 (A-10680, Thermo Scientific); Goat anti-Mouse IgM (Heavy chain), Alexa Fluor® 594 (A-21044, Thermo Scientific); Donkey anti-Mouse IgG (H+L), Alexa Fluor® 594 (A-21203, Thermo Scientific); Donkey anti-Mouse IgG (H+L), Alexa Fluor® 488 (A-21202, Thermo Scientific); Donkey anti-Rabbit IgG (H+L), Alexa Fluor® 594 (A-21207, Thermo Scientific); Donkey anti-Rabbit IgG (H+L), Alexa Fluor® 488 (A-21206, Thermo Scientific).

APPENDIX FIGURE LEGENDS

Appendix Figure S1. Transcriptional stress inhibits rRNA synthesis (related to Figure 2 and EV2). Approximate location of the qPCR primers is shown: 45S, 18S 5'J (5' Junction); 18S; 5.8S 5'J (5' Junction); 5.8S; 28S (from left to right). RT-qPCR analysis of the expression levels of precursor and mature ribosomal RNAs in HeLa cells treated for 6 hrs as described (MG132 10 µM; Act.D 4 µM; CHX 50 µg/ml). Statistical significance via One-way ANOVA; n.s. = non-significant; n = 3 independent experiments, +/- s.e.m.

Appendix Figure S2. Upon temperature upshift, the model substrate NLuc-GFP accumulates in nucleoli and PML-NBs (related to Figure 3). HeLa cells were lipofected with cDNAs encoding for NLuc-GFP. 24 h post-transfection, cells were left untreated (Control) or treated with HS at 42˚C for 2 h, alone or with CHX (50 µg/ml). When stated, cells recovered during 3 h after HS. Cells were fixed and nuclei were stained with DAPI. * indicate nucleoli. Scale bars: 5 µm.

Appendix Figure S3. Upon proteasome inhibition or temperature upshift, HSPA1A, HSPA8 and VCP are recruited to PML-NBs enriched for newly synthesized aberrant proteins (related to Figure 5). (A-C) Labelling of PML, HSPA1A, HSPA8, VCP or DRiPs in HeLa cells left untreated or treated with MG132 (10 µM) and OP-puro (25 µM) for 4 h. **(D)** Labelling of HSPA1A, HSPA8 or VCP in PML-GFP HeLa Kyoto cells treated with OP-puro (25 µM) and heat shock (HS) at 42˚C for 2 h. **(E)** Labelling of DRiPs and polyUb proteins (FK1) in HeLa cells treated with MG132 (10 µM) and OP-puro (25 µM) for 4 hrs, followed by recovery in drug-free medium (control) or with VER (40 µM) for 5 hrs. Scale bars: 10 µm.

APPENDIX FIGURE REFERENCES

Kwon I, Xiang S, Kato M, Wu L, Theodoropoulos P, Wang T, Kim J, Yun J, Xie Y, McKnight SL (2014) Poly-dipeptides encoded by the C9orf72 repeats bind nucleoli, impede RNA biogenesis, and kill cells. *Science* 345: 1139-45

Morelli FF, Verbeek DS, Bertacchini J, Vinet J, Mediani L, Marmiroli S, Cenacchi G, Nasi M, De Biasi S, Brunsting JF, Lammerding J, Pegoraro E, Angelini C, Tupler R, Alberti S, Carra S (2017) Aberrant Compartment Formation by HSPB2 Mislocalizes Lamin A and Compromises Nuclear Integrity and Function. *Cell reports* 20: 2100-2115

Nishimura K, Kumazawa T, Kuroda T, Katagiri N, Tsuchiya M, Goto N, Furumai R, Murayama A, Yanagisawa J, Kimura K (2015) Perturbation of ribosome biogenesis drives cells into senescence through 5S RNP-mediated p53 activation. *Cell reports* 10: 1310-23

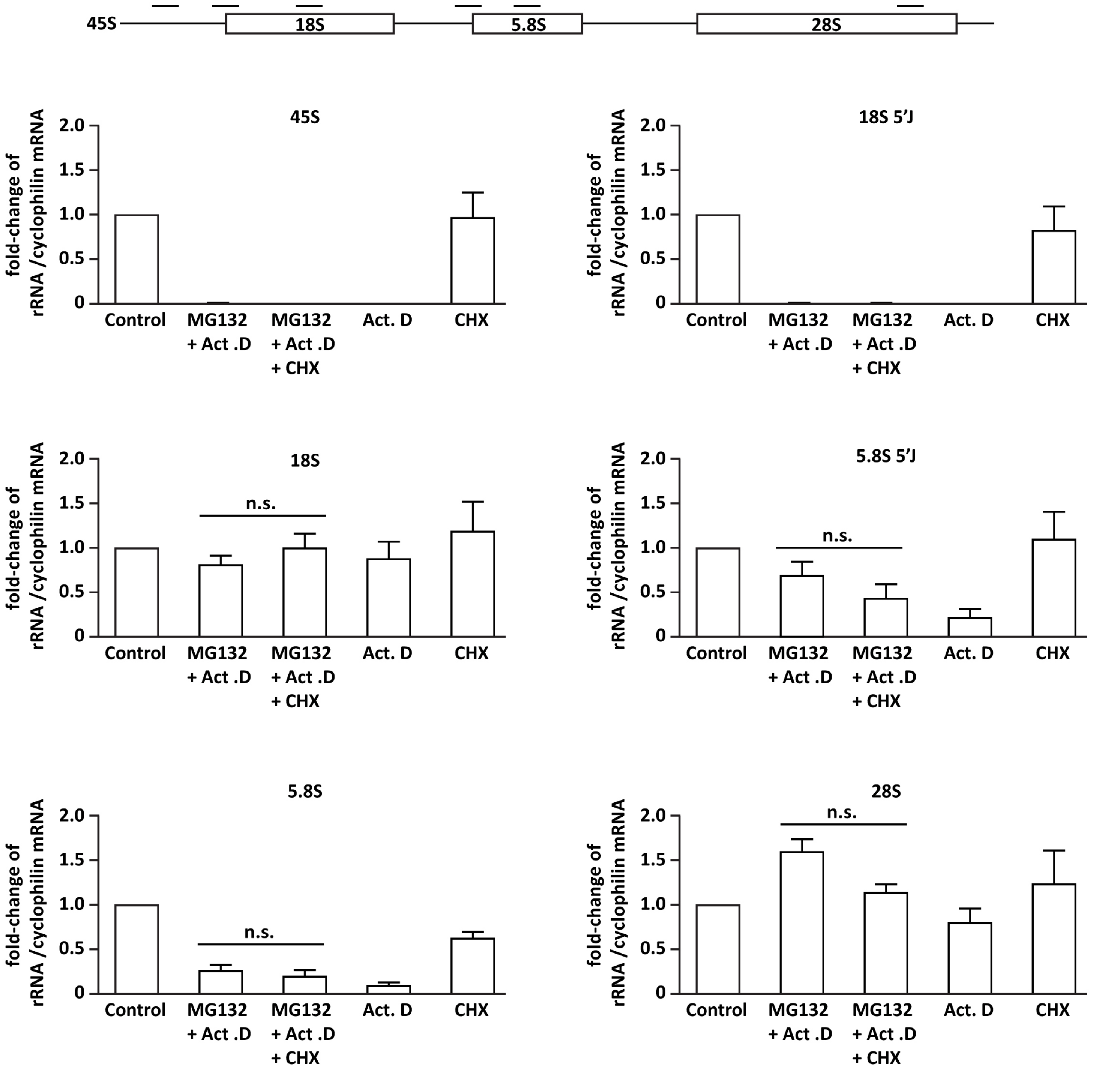

Appendix Figure S1. Transcriptional stress inhibits rRNA synthesis (related to Figure 2 and EV2). Approximate location of the qPCR primers is shown: 45S, 18S 5'J (5' Junction); 18S; 5.8S 5'J (5' Junction); 5.8S; 28S (from left to right). RT-qPCR analysis of the expression levels of precursor and mature ribosomal RNAs in HeLa cells treated for 6 hrs as described (MG132 10 μM; Act.D 4 μM; CHX 50 μg/ml). Statistical significance via One-way ANOVA; n.s. = non-significant; n = 3 independent experiments, +/- s.e.m.

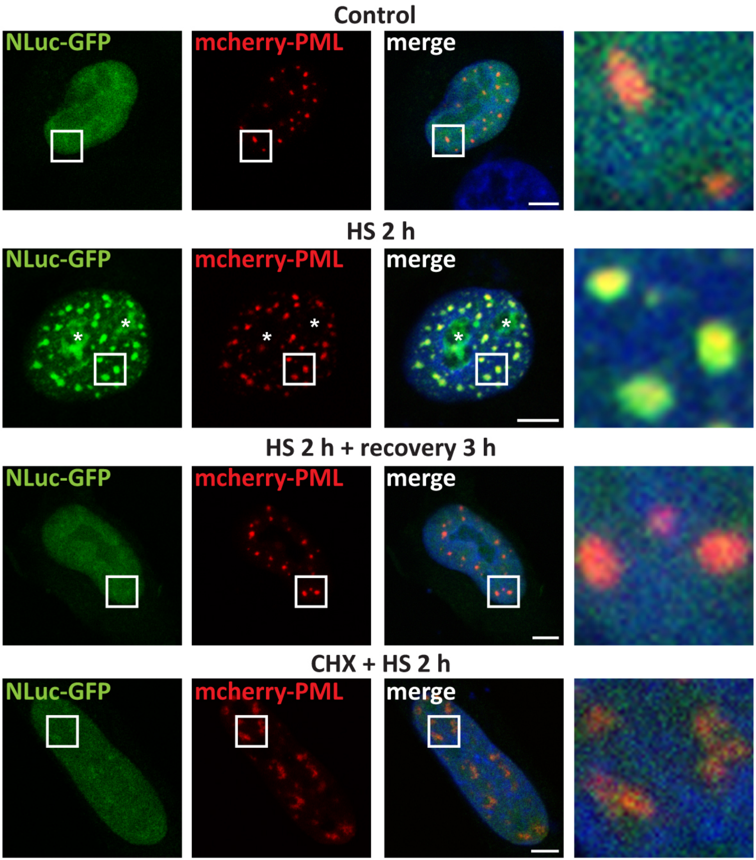

Appendix Figure S2. Upon temperature upshift, the model substrate NLuc-GFP accumulates in nucleoli and PML-NBs (related to Figure 3). HeLa cells were lipofected with cDNAs encoding for NLuc-GFP and mCherry-PML. 24 h post-transfection, cells were left untreated (Control) or treated with HS at 42˚C for 2 h, alone or with CHX (50 µg/ml). When stated, cells recovered during 3 h after HS. Cells were fixed and nuclei were stained with DAPI. * indicate nucleoli. Scale bars: 5 µm.

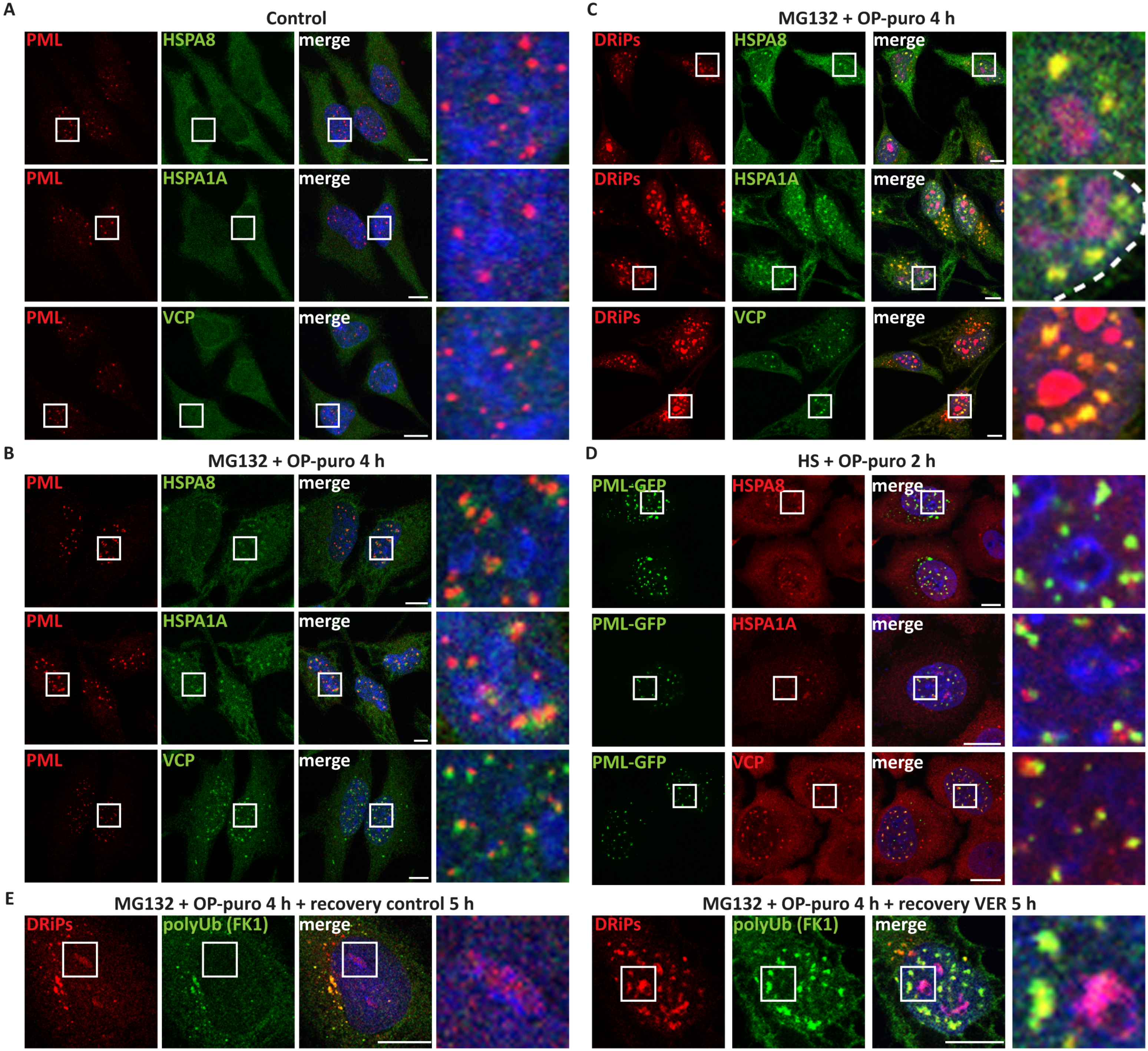

Appendix Figure S3. Upon proteasome inhibition or temperature upshift, HSPA1A, HSPA8 and VCP are recruited to PML-NBs enriched for newly synthesized aberrant proteins (related to Figure 5). (A-C) Labelling of PML, HSPA1A, HSPA8, VCP or DRiPs in HeLa cells left untreated or treated with MG132 (10 µM) and OP-puro (25 µM) for 4 h. **(D)** Labelling of HSPA1A, HSPA8 or VCP in PML-GFP HeLa Kyoto cells treated with OP-puro (25 µM) and heat shock (HS) at 42˚C for 2 h. **(E)** Labelling of DRiPs and polyUb proteins (FK1) in HeLa cells treated with MG132 (10 µM) and OP-puro (25 µM) for 4 hrs, followed by recovery in drug-free medium (control) or with VER (40 µM) for 5 hrs. Scale bars: 10 µm.
