# Supplementary material for: Defective ribosomal products challenge nuclear function by impairing nuclear condensate dynamics and immobilizing ubiquitin
Source: EMBO J. 2019 Jul 4;38(15):e101341. doi: 10.15252/embj.2018101341 (PMC6669919; doi:10.15252/embj.2018101341)
Supplement: Supplementary file 2 — Expanded View Figures PDF [file EMBJ-38-e101341-s002.pdf]

Expanded View Figures

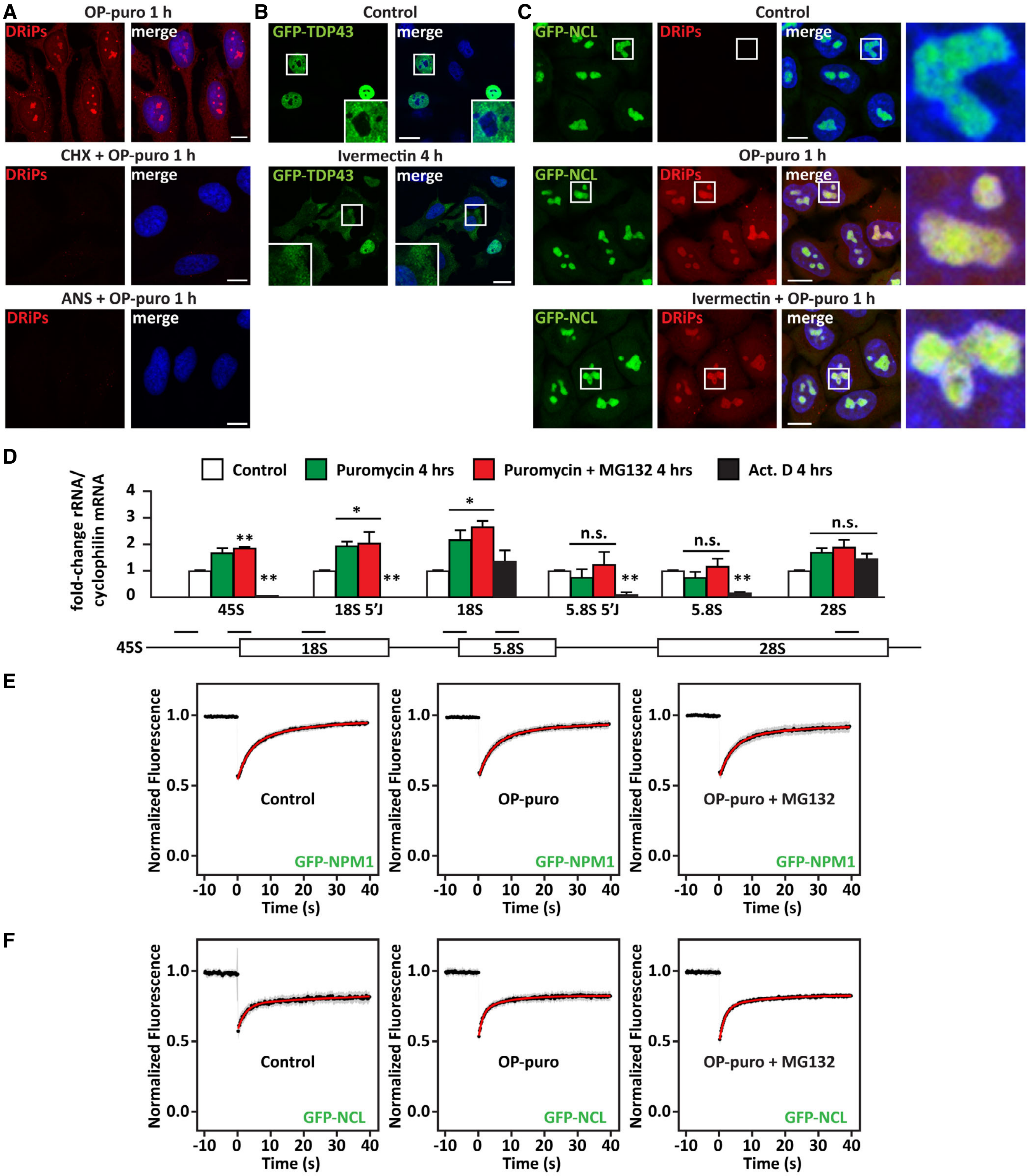

Figure EV1.

 © 2019 The Authors

Figure EV1. DRiPs accumulate in subnucleolar compartments, without affecting the dynamics of NCL and NPM1 (related to Fig 1).

A DRiP labeling in HeLa cells that were treated with OP-puro (25 μM) alone or combined with the translation inhibitors CHX (50 μg/ml) and anisomycin (ANS; 5 μg/ml). Scale bars: 10 μm.

B Hela cells overexpressing GFP-TDP43 were left untreated or treated with the nuclear import inhibitor ivermectin (25 μM) for 4 h, prior to fixation and imaging. Scale bars: 20 μm.

C DRiP labeling in GFP-NCL HeLa Kyoto cells that were left untreated (Control) or treated with OP-puro (25 μM) alone or combined with Ivermectin (25 μM). Scale bars: 10 μm.

D RT–qPCR analysis of the expression levels of precursor and mature ribosomal RNAs in HeLa cells treated as described (MG132 10 μM; puromycin 10 μg/μl; actinomycin D/Act.D 4 μM). Statistical significance via one-way ANOVA; $*P = 0.01$; $**P = 0.001$; $n = 3$ independent experiments, ± s.e.m. Approximate location of the qPCR primers is shown: 45S, 18S 5′J (5′ Junction); 18S; 5.8S 5′J (5′ Junction); and 5.8S; 28S (from left to right).

E, F Quantitation of the fluorescence intensity recovery after photobleaching (FRAP) of GFP-NPM1 (E) and GFP-NCL (F). Cells were left untreated, or treated with OP-puro alone (25 μM), or combined with MG132 (20 μM). The average recovery curve ($N = 17$–22) is shown in black, while the fitting curve is shown in red. Gray shows the SD.

© 2019 The Authors

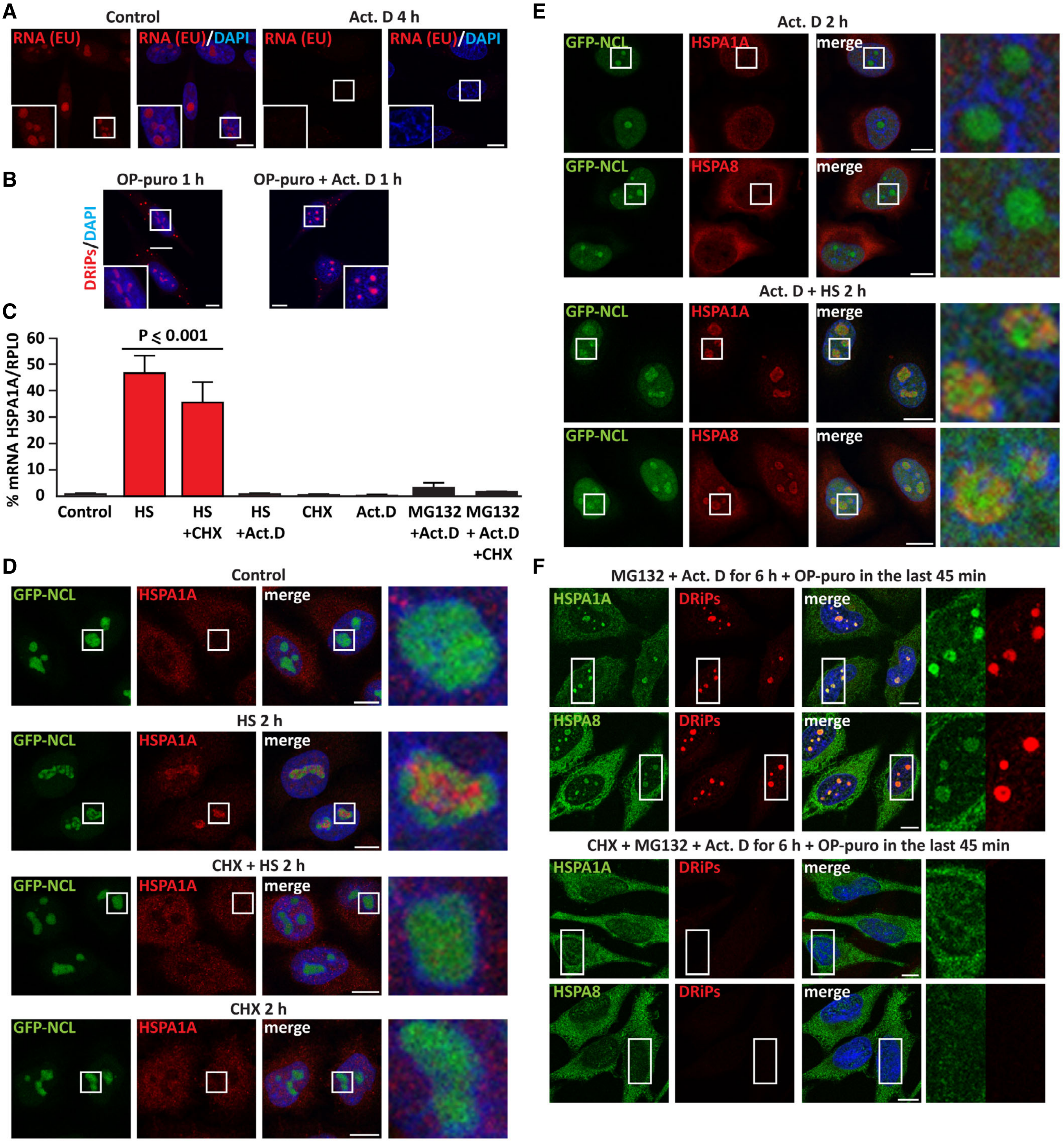

Figure EV2.

 © 2019 The Authors

Figure EV2. Upon stress, inhibition of translation prevents the recruitment of HSPA1A and HSPA8 inside nucleoli (related to Fig 2).

A Staining of newly synthesized RNA with 5-ethynyl uridine (EU, 200 μM) alone or with actinomycin D (Act. D; 4 μM) for 4 h, followed by click chemistry. Nucleic acid is stained with DAPI.
B DRiP labeling in HeLa cells treated with OP-puro (25 μM) alone or combined with Act. D (4 μM) for 1 h.
C HeLa cells were either left untreated (Control) or treated as follows: HS at 42°C for 3 h (HS), alone or combined with CHX (50 μg/ml) or Act. D (3 μM); single CHX or Act. D treatment for 3 h; transcriptional stress for 6 h (MG132 10 μM with Act. D 4 μM) (Audas *et al*, 2016), alone or combined with CHX (50 μg/ml). After cellular treatment, total RNA was extracted and mRNA levels of HSPA1A were measured by RT–qPCR (RPL0 was used for normalization). Statistical significance via one-way ANOVA; n = 3, ± s.e.m.
D GFP-NCL HeLa Kyoto cells were left untreated or exposed to HS at 42°C for 2 h alone or with CHX; as control, cells were also treated with CHX alone for 2 h (50 μg/ml). Cells were then fixed and stained for HSPA1A and DAPI.
E Staining of HSPA1A and HSPA8 in GFP-NCL HeLa Kyoto cells treated with Act. D (3 μM) alone or combined with HS at 42°C for 2 h.
F Staining of HSPA1A, HSPA8, and DRiPs in HeLa cells subjected to transcriptional stress (MG132 10 μM with Act. D 4 μM) alone or concomitant to translation inhibition (CHX 50 μg/ml) for 6 h. OP-puro (25 μM) was added during the last 45 min of treatment.

Data information: (A, B, D-F): Scale bars: 10 μm.

Figure EV3. DRiPs that accumulate upon proteasome inhibition or temperature upshift are ubiquitinated and do not colocalize with nuclear speckles (related to Fig 3).

A Subcellular distribution of DRiPs and SC35, used as a marker for nuclear speckles, in HeLa cells that were left untreated or treated as indicated. Scale bars: 10 μm.
B Immunoprecipitation (IP) of puromycylated proteins from nucleolar or nucleoplasmic extracts. HeLa cells were treated as indicated for 2 h (25 μM puromycin, 20 μM MG132) before fractionation. Western blots against puromycin and ubiquitin are shown.
C HeLa cells were treated with puromycin (5 μg/ml) for 1 h, followed by recovery for 6 h in drug-free medium (−) or in the presence of MG132 (10 μM) or NH_4Cl (20 mM). Cytoplasmic and nuclear proteins were fractionated. Clearance of puromycylated and ubiquitinated proteins were analyzed by immunoblotting in both fractions. TUBA4A and LMNB1 were used as cytoplasmic and nuclear loading controls.

© 2019 The Authors

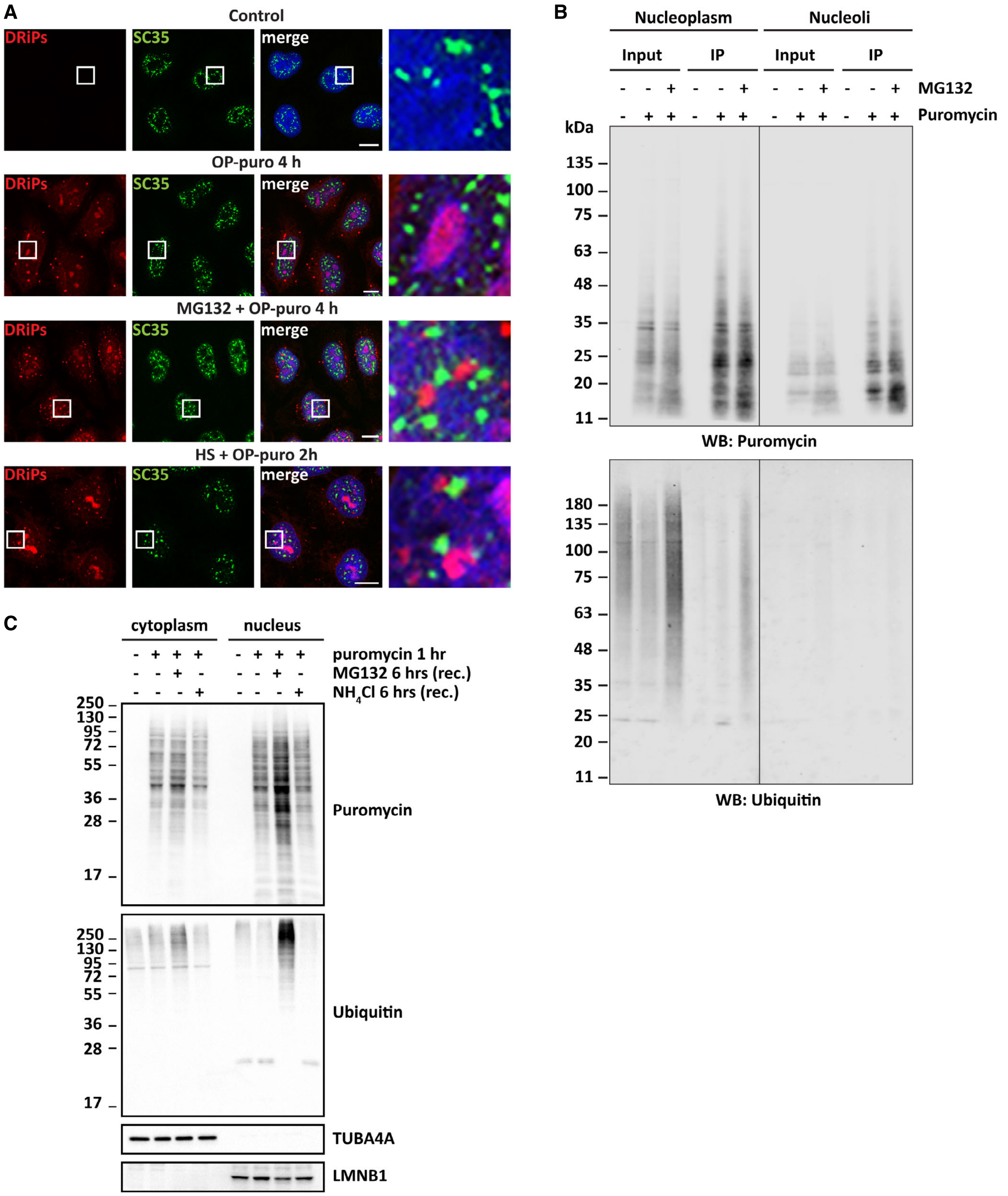

Figure EV3.

 © 2019 The Authors

 © 2019 The Authors

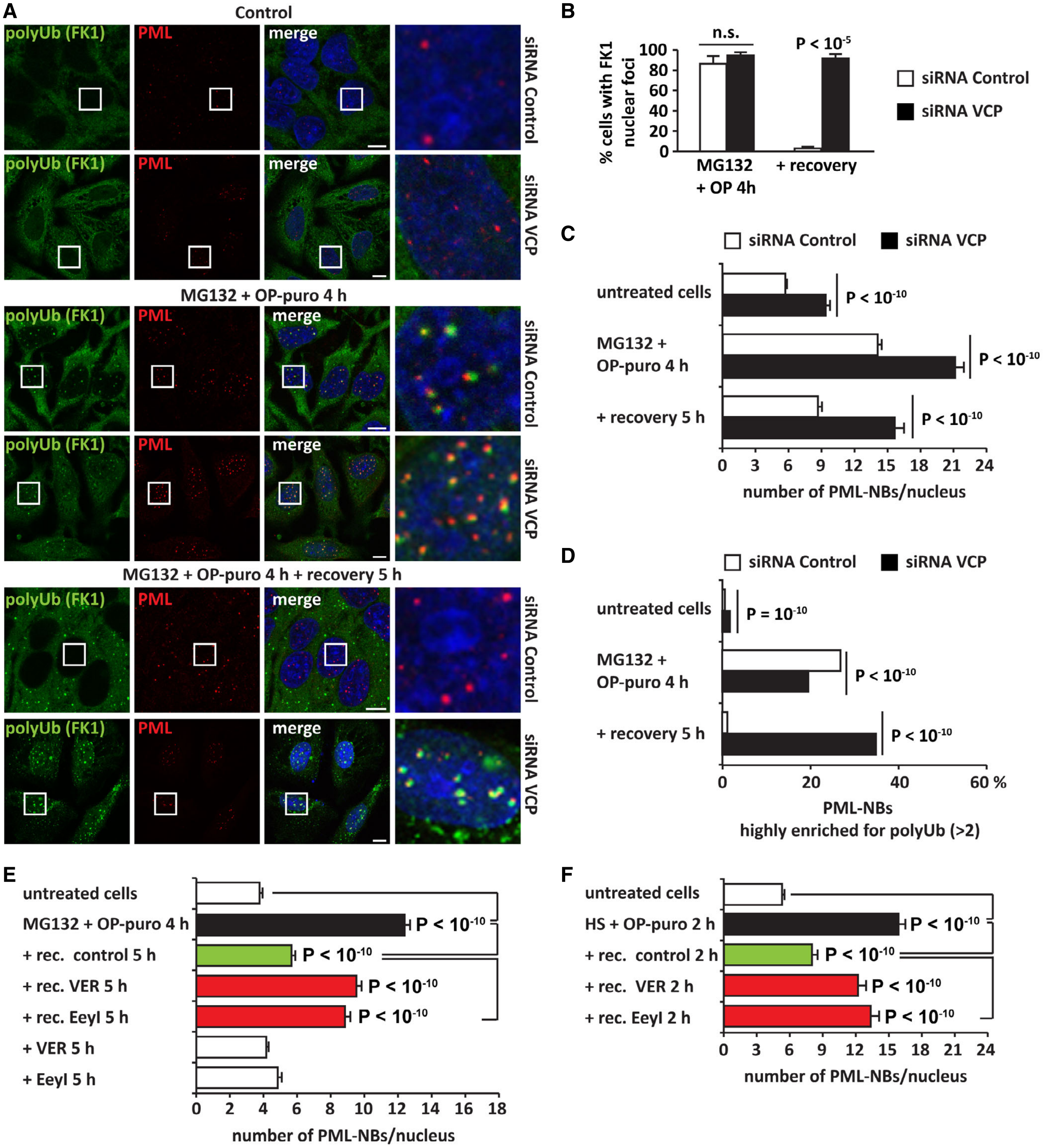
A
Control
polyUb (FK1)
PML
merge
siRNA Control
siRNA VCP
MG132 + OP-puro 4 h
MG132 + OP-puro 4 h + recovery 5 h
B
% cells with FK1 nuclear foci
n.s.
P < 10-5
MG132 + OP 4h
+ recovery
siRNA Control
siRNA VCP
C
untreated cells
MG132 + OP-puro 4 h
+ recovery 5 h
P < 10-10
number of PML-NBs/nucleus
D
P = 10-10
PML-NBs highly enriched for polyUb (>2)
E
untreated cells
MG132 + OP-puro 4 h
+ rec. control 5 h
+ rec. VER 5 h
+ rec. EeyI 5 h
+ VER 5 h
+ EeyI 5 h
number of PML-NBs/nucleus
F
untreated cells
HS + OP-puro 2 h
+ rec. control 2 h
+ rec. VER 2 h
+ rec. EeyI 2 h
number of PML-NBs/nucleus

Figure EV4.

Figure EV4. VCP depletion delays the clearance of DRiPs and polyUb proteins from PML-NBs (related to Figs 5 and 6).

A HeLa cells were lipofected with non-targeting siRNA control or a specific siRNA against VCP. 72 h post-transfection, cells were either left untreated, or treated with MG132 (10 μM) and OP-puro (25 μM) for 4 h; where indicated, cells were allowed to recover in drug-free medium for 5 h. Cells were fixed and processed for immunostaining of PML and polyUb proteins (FK1). Scale bars: 10 μm.

B Quantitation of cells shown in (A). Number of cells counted/condition: 169–1,730 in three independent experiments; statistical significance via one-way ANOVA; $P < 10^{-5}$, ± s.e.m.

C, D Automated PML-NB segmentation is based on PML signal. (C) Quantitation of the number of PML-NBs/nucleus of cells shown in (A). n = 130–1,109; $P < 10^{-10}$. (D) Quantitation of the number of PML-NBs enriched for polyUb (> 2); n = 2,052–8,452; statistical significance via one-way ANOVA; $P < 10^{-10}$.

E, F PML are stress-responsive membraneless organelles whose number changes in response to proteasome inhibition, heat shock, and accumulation of DRiPs. HeLa cells were left untreated or treated as described in the graphics (for drug concentration, see Fig 5). Quantitation of the number of PML-NBs/nucleus of cells is shown. Automated PML-NB segmentation is based on PML signal. (E) Statistical significance via one-way ANOVA. n = 231–644; $P < 10^{-10}$, ± s.e.m. (F) n = 142–312 $P < 10^{-10}$, ± s.e.m.

 © 2019 The Authors

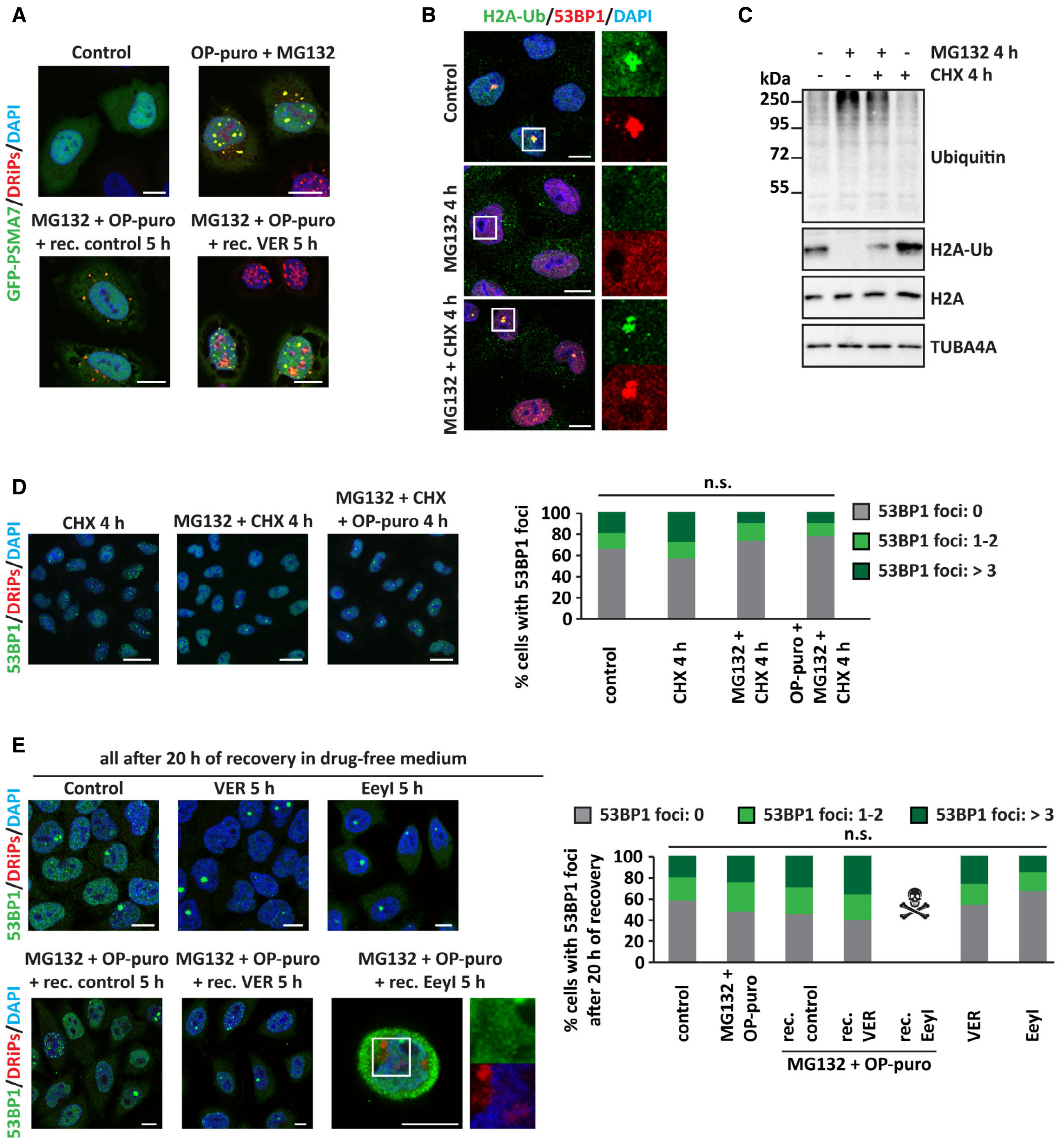
A
Control
OP-puro + MG132
MG132 + OP-puro + rec. control 5 h
MG132 + OP-puro + rec. VER 5 h
GFP-PSMA7/DRiPs/DAPI
B
H2A-Ub/53BP1/DAPI
Control
MG132 4 h
MG132 + CHX 4 h
C
MG132 4 h
CHX 4 h
kDa
250
95
72
55
Ubiquitin
H2A-Ub
H2A
TUBA4A
D
CHX 4 h
MG132 + CHX 4 h
MG132 + CHX + OP-puro 4 h
53BP1/DRiPs/DAPI
n.s.
% cells with 53BP1 foci
53BP1 foci: 0
53BP1 foci: 1-2
53BP1 foci: > 3
control
CHX 4 h
MG132 + CHX 4 h
OP-puro + MG132 + CHX 4 h
E
all after 20 h of recovery in drug-free medium
Control
VER 5 h
Eeyl 5 h
MG132 + OP-puro + rec. control 5 h
MG132 + OP-puro + rec. VER 5 h
MG132 + OP-puro + rec. Eeyl 5 h
53BP1/DRiPs/DAPI
% cells with 53BP1 foci after 20 h of recovery
n.s.
control
MG132 + OP-puro
rec. control
rec. VER
rec. Eeyl
VER
Eeyl
MG132 + OP-puro

Figure EV5.

Figure EV5. Solid PML-NBs sequester 20S proteasomes and compromise H2A ubiquitination and 53BP1 foci formation (related to Fig 7).

A GFP-PSMA7 HeLa Kyoto cells were left untreated or treated as described. Cells were fixed and subjected to click chemistry to visualize DRiPs. Nucleic acid was stained with DAPI. Scale bars: 10 μm.

B, C Hela cells were left untreated or treated for 4 h with MG132 (10 μM), alone or combined with CHX (50 μg/ml). (B) Cells were fixed and processed for immunostaining of H2A-Ub and 53BP1. Scale bars: 10 μm. (C) Expression levels of ubiquitin, H2A-Ub, and H2A in total protein extracts. TUBA4A was used as loading control.

D HeLa cells were left untreated or treated for 4 h with CHX, MG132, OP-puro, combined as indicated and using the concentrations previously reported. Representative pictures of 53BP1 distribution and quantitation of the % of cells with 53BP1 foci/nucleus are shown. Number of cells counted/condition: 681–1,214 in three independent experiments; statistical significance via one-way ANOVA; *P* = not significant (n.s.). Scale bars: 20 μm.

E Cells treated as described, using the concentrations previously reported, were allowed to recover in drug-free medium for 20 h. Cells were then fixed, and 53BP1 and DRiPs were stained. Quantitation of the % of cells with 53BP1 foci/nucleus is shown. Number of cells counted/condition: 668–1,678 in three independent experiments; statistical significance via one-way ANOVA; *P* = not significant (n.s.). Scale bars: 10 μm.

 © 2019 The Authors
