# Supplementary material for: Defective ribosomal products challenge nuclear function by impairing nuclear condensate dynamics and immobilizing ubiquitin
Source: EMBO J. 2019 Jul 4;38(15):e101341. doi: 10.15252/embj.2018101341 (PMC6669919; doi:10.15252/embj.2018101341)
Supplement: Supplementary file 3 — Movie EV1 [file EMBJ-38-e101341-s003.zip › Movie_EV1.docx]

**Movie EV1: mCherry-VHL is not recruited inside PML-GFP bodies in normally growing HeLa cells.**

Related to Figure 6
