# Supplementary material for: Defective ribosomal products challenge nuclear function by impairing nuclear condensate dynamics and immobilizing ubiquitin
Source: EMBO J. 2019 Jul 4;38(15):e101341. doi: 10.15252/embj.2018101341 (PMC6669919; doi:10.15252/embj.2018101341)
Supplement: Supplementary file 5 — Movie EV3 [file EMBJ-38-e101341-s005.zip › Movie_EV3.docx]

**Movie EV3: During the stress recovery phase, mCherry-VHL is cleared from PML-GFP bodies.**

Related to Figure 6
