# Supplementary material for: Defective ribosomal products challenge nuclear function by impairing nuclear condensate dynamics and immobilizing ubiquitin
Source: EMBO J. 2019 Jul 4;38(15):e101341. doi: 10.15252/embj.2018101341 (PMC6669919; doi:10.15252/embj.2018101341)
Supplement: Supplementary file 10 — Movie EV8 [file EMBJ-38-e101341-s010.zip › Movie_EV8.docx]

**Movie EV8: GFP-PSMA7 and mCherry-VHL are cleared from nuclear bodies** **during the stress recovery phase.**

Related to Figure 7
