# Supplementary material for: Defective ribosomal products challenge nuclear function by impairing nuclear condensate dynamics and immobilizing ubiquitin
Source: EMBO J. 2019 Jul 4;38(15):e101341. doi: 10.15252/embj.2018101341 (PMC6669919; doi:10.15252/embj.2018101341)
Supplement: Supplementary file 11 — Movie EV9 [file EMBJ-38-e101341-s011.zip › Movie_EV9.docx]

**Movie EV9: Inhibition of HSP70, during the stress recovery phase, prevents the clearance of GFP-PSMA7 and mCherry-VHL from nuclear bodies.**

Related to Figure 7
